# Magnetotransport Signatures of Spin–Orbit Coupling in High‐Temperature Cuprate Superconductors

**DOI:** 10.1002/advs.76166

**Published:** 2026-07-17

**Authors:** Aleix Barrera, Huidong Li, Thomas Gunkel, Jordi Alcalá, Silvia Damerio, Can Onur Avci, Anna Palau

**Affiliations:** ^1^ Insititut de Ciència de Materials de Barcelona (ICMAB‐CSIC) Cerdanyola del Vallès Spain

**Keywords:** anisotropic magnetoresistance, high‐temperature superconductors, planar Hall effect, quasiparticles, superconducting spintronics, spin–orbit coupling

## Abstract

Spin transport in superconductors offers a compelling platform to merge the dissipationless nature of superconductivity with the functional promise of spin‐based electronics. A significant challenge in achieving spin polarization in conventional superconductors stems from the singlet state of Cooper pairs, which exhibit no net spin. The generation of spin‐polarized carriers, quasiparticles, or triplet pairs in superconductors has predominantly been realized in hybrid superconductor/ferromagnet systems through proximity‐induced spin polarization. Historically, cuprate superconductors have been characterized by strong electronic correlations but negligible spin–orbit coupling. In this study, we observe a large in‐plane angle‐dependent magnetoresistance and a pronounced planar Hall effect arising near the superconducting phase transition in the prototypical high‐temperature cuprate superconductor $YBa_{2}Cu_{3}O_{7-x}$ without using a proximity ferromagnet. These effects ‐ unusual in centrosymmetric cuprates ‐ may arise from spin‐polarized quasiparticle transport potentially mediated by strong spin–orbit coupling. By systematically tuning magnetic field strength, orientation, temperature, and doping, we identify transport signatures that are consistent with spin–orbit‐driven phenomena. Our findings suggest the presence of a previously underappreciated spin–orbit landscape in cuprates, which may provide the basis for exploring spintronic functionalities in high‐temperature superconductors.

## Graphical abstract

Large angle‐dependent magnetoresistance and planar Hall effects emerge at the superconducting transition of ${\mathrm{YBa}}_{2}{\mathrm{Cu}}_{3}O_{7-x}$ thin films. These anomalous transport signatures, absent in the normal state, point to spin‐polarized quasiparticle dynamics enabled by an unexpected spin–orbit coupling landscape in cuprate superconductors, opening new routes toward high‐temperature superconducting spintronics.

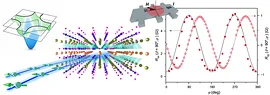

## 1 Introduction

Unusual magnetotransport phenomena at phase transitions provide a powerful lens for exploring the intricate interplay between charge, spin, and lattice degrees of freedom in condensed matter systems. Classic examples include colossal magnetoresistance in correlated oxides [1], magnetocaloric effects in rare‐earth alloys [2], quantum Hall transitions in two‐dimensional electron gases [3], and topological phase transitions in Weyl semimetals [4]. More recently, novel spin transport effects have emerged at magnetic phase transitions, such as spin colossal magnetoresistance [5] and longitudinal spin pumping [6]. These phenomena illustrate the dynamic evolution of spin and charge in response to symmetry breaking and the changes in the order parameters, revealing emergent physical effects that govern electronic transport. Unraveling these mechanisms deepens our understanding of fundamental physics and holds promise for next‐generation technologies in energy‐efficient electronics, quantum materials, and spintronic computing.

Spin transport in superconducting materials represents a particularly intriguing frontier, as it combines the dissipationless nature of superconductivity with the potential advantages of spin‐based electronics. This field offers exciting opportunities for energy‐efficient information processing, storage, and transmission [7, 8]. However, generating and manipulating spin polarization in conventional superconductors is inherently challenging due to the singlet nature of Cooper pairs, which carry zero net spin. Beyond the realm of triplet superconductivity, spin‐polarized quasiparticles have emerged as a promising alternative, exhibiting distinct transport properties that differ from those of conventional electrons. In particular, quasiparticle‐mediated spin currents could surpass electron‐mediated currents in efficiency, owing to their longer spin lifetimes and the possibility of spin–charge separation, enabling nearly dissipationless transport [9, 10, 11]. Furthermore, quasiparticle‐mediated spin Hall effects may induce giant spin‐to‐charge conversion phenomena [12, 13]. Notably, in the presence of an in‐plane magnetic field, spin‐polarized quasiparticles can emerge even from a non‐magnetic injector due to Zeeman splitting of the density of states [10, 14, 15].

Magnetoresistance and Hall effects provide invaluable tools for probing the electronic properties of complex quantum materials. In superconductors, these techniques serve as sensitive probes of the interplay between competing interactions, often revealing unexpected transport anomalies [16]. For instance, the Anomalous Hall Effect (AHE) in superconductors may arise from multiple mechanisms, including vortex motion with pinning, superconducting fluctuations [17, 18], quasiparticle excitations, or topological scattering effects [19, 20]. In cuprate superconductors, spontaneous transverse voltages have been observed even at zero applied field, suggesting a possible intrinsic electronic nematicity [21, 22, 23]. Additionally, anisotropic magnetoresistance (AMR) and its transverse counterpart, the planar Hall effect (PHE), have been proposed as probes of hidden spin symmetries and exotic electronic states driven by spin–orbit coupling (SOC) and topological band structure effects [24, 25]. These transport signatures may offer a powerful means to uncover previously hidden physics, particularly in the context of unconventional superconductivity and emergent spin–orbit interactions.

In this study, we observe an exceptionally large in‐plane angle‐dependent magnetoresistance (ADMR) and planar Hall effects occurring within a narrow temperature window at the superconducting phase transition in the archetypal high‐temperature cuprate superconductor ${\mathrm{YBa}}_{2}{\mathrm{Cu}}_{3}O_{7-x}$ (YBCO). Systematic measurements as a function of external magnetic field, temperature, oxygen doping level, and field geometry reveal transport features that are consistent with spin‐polarized quasiparticle‐mediated transport, which may reflect underlying SOC interactions. These results raise the possibility of SOC‐related effects in centrosymmetric cuprate superconductors, a scenario that has been traditionally neglected. Despite being overlooked until now, recent measurements using spin‐ and angle‐resolved spectroscopy have uncovered interesting signs of SOC effects in bismuth‐based superconductors [26, 27, 28]. Building on these findings, theoretical predictions suggest that various spin–orbit‐driven phenomena could occur in cuprate superconductors [29]. However, direct transport measurements reflecting these effects were missing. Our findings contribute to this evolving picture by providing new experimental insights into charge and spin transport in high–temperature superconductors. The transport phenomena we observe are consistent across films with different thicknesses and doping levels, supporting the presence of a significant intrinsic component. At the same time, the magnitude and temperature dependence of the signals may be influenced by extrinsic factors, including disorder, strain, and variations in oxygen content introduced during thinfilm fabrication. These observations point to spin–dependent transport mechanisms that may be relevant for spin–based electronic devices operating at liquid nitrogen temperatures. However, translating these phenomena into functional devices will require a rigorous disentanglement and quantitative assessment of intrinsic electronic mechanisms relative to fabrication–related effects, together with stringent control of materials and interface quality. In particular, the fragility of oxide interfaces, their sensitivity to disorder and oxygen non–stoichiometry, and the difficulty of preserving intrinsic electronic properties during device processing impose critical constraints on the scalability and reproducibility of thin–film cuprate devices, including emerging cuprate twistronics architectures [30, 31].

## 2 Sample Description and Planar Hall Effect Measurements

**FIGURE 1:**
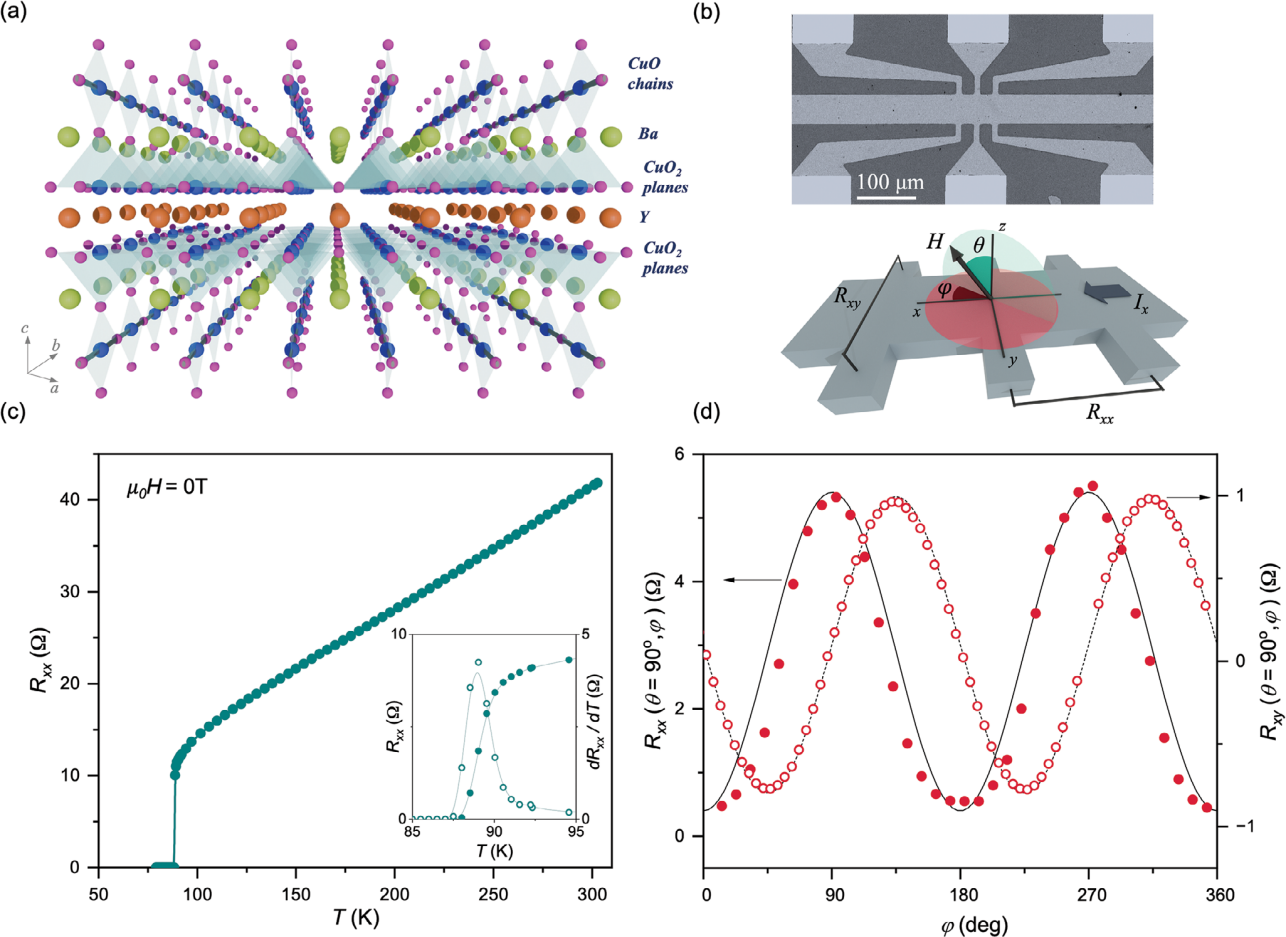
Layer structure, device layout, and magnetotransport measurements. (a) Crystal structure of YBCO. (b) Scanning electron micrograph of a sample (top) and schematics of the angular longitudinal and transversal resistance measurements (bottom). (c) Temperature dependence of the longitudinal magnetoresistance component measured at zero applied field for a typical YBCO device of 50nm. Inset shows a zoom of the magnetoresistance (closed symbols, left axis) and its first derivative (open symbols, right axis). (d) In‐plane angular dependence of the magnetoresistance (closed symbols, left axis) and Hall resistance (open symbols, right axis) measured at 88K and 8T. Solid and dashed lines show the angular dependence of $\cos^{2}(\varphi$) and sin(φ)cos(φ), respectively.

Epitaxial ${\mathrm{YBa}}_{2}{\mathrm{Cu}}_{3}O_{7-x}$ (YBCO) superconducting thin films were grown by pulsed laser deposition on single crystal substrates (details in Methods). YBCO is a layered perovskite high‐temperature superconductor with an orthorhombic crystal structure, where superconductivity arises primarily from the ${\mathrm{CuO}}_{2}$ bilayers separated by Y and Ba spacer layers. The additional CuO chain layers along the *b*‐axis are crucial in charge doping and transport, influencing the anisotropic electronic properties of the material (Figure 1a). The superconducting transition temperature and electronic phase diagram are susceptible to oxygen stoichiometry, with optimal doping *x* = 0.08 yielding $T_{c}$ = 93 K [32]. Films are patterned in Hall bar structures with a length *l* = 20–30 μm, width w = 20–50 μm, as shown in the top panel of Figure 1b. Hall effect, $R_{xy}$, and magnetoresistance measurements, $R_{xx}$, described below, are performed with electrical connections illustrated in the lower panel of Figure 1b. The orientation of the applied magnetic field relative to the current direction is defined by the angles θ and φ, as also indicated in Figure 1b.

Figure 1c depicts the temperature dependence of the longitudinal resistance ($R_{xx}$) measured in the absence of an external magnetic field for an optimally doped 50 nm‐thick YBCO. The superconducting transition is determined at the first derivative's maximum and reads $T_{c}$ = 89 K, which is slightly below the bulk value and indicates an excellent film quality. We next measure $R_{xx}$ and its transverse counterpart $R_{xy}$ at 88 K, i.e., at the onset of superconductivity, by rotating the magnetic field within the *xy*‐plane at an amplitude of 8 T. The results are shown in Figure 1d and represent the most critical findings of this study. We find an exceptionally large in‐plane ADMR, $R_{xx}(\theta =90^{\circ },\varphi$), exceeding 1000% (Figure 3b) and a sizeable PHE resistance, $R_{xy}(\theta =90^{\circ },\varphi$) ‐ the transverse counterpart of ADMR, close to 1 Ohm. Both signals follow the characteristic symmetries typically observed for AMR and PHE in ferromagnetic materials, i.e., ∝ $\cos^{2}(\varphi$) and ∝ sin(φ)cos(φ), respectively, despite the absence of ferromagnetic ordering in YBCO.

**FIGURE 2:**
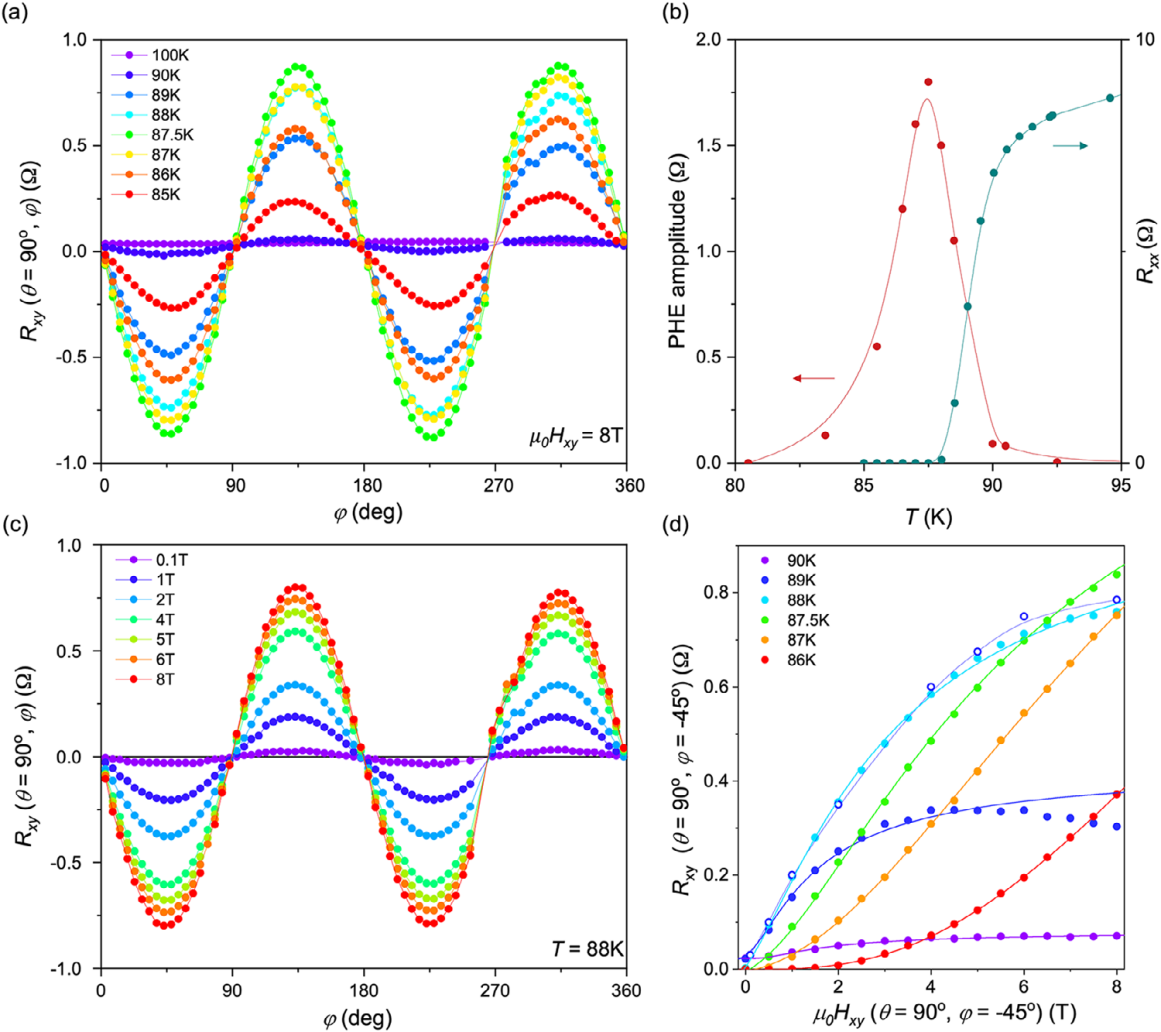
Angle‐dependent Hall resistances. (a,c) Angular dependence of the planar Hall resistance, $R_{xy}\left(\varphi \right)$ measured at 8 T for different temperatures (a) and 88 K for different applied magnetic fields (c) for a typical YBCO device of 50 nm. (b) Red closed symbols show the temperature dependence of the PHE amplitude obtained by fitting the curves in (a) with a sin(φ)cos(φ) dependence. Cyan open symbols show the longitudinal magnetoresistance at zero magnetic field, plotted at the right axis. (d) Magnetic field dependence of $R_{xy}$ at φ = 135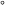. Open symbols show half of the PHE amplitude obtained by fitting the curves in **c** with a sin(φ)cos(φ) dependence. Solid lines show fits to $n_{QP}/\left(1+exp\left(H_{1}/\left(H+H_{0}\right)\right)\right)$.

Next, we examine the temperature and field dependence of PHE to better understand its origins and extent. In Figure 2a, we plot the PHE signal as a function of temperature through the superconducting transition window (85–90 K) and at 100 K as a reference. PHE is virtually absent in the normal state (100 K) and appears only when YBCO transitions to the superconducting state, with the largest signal recorded at 87.5 K. Figure 2b shows the temperature dependence of PHE (left axis) plotted together with $R_{xx}$ (right axis). The onset of large PHE coincides with the onset of the superconducting transition, while the peak value occurs precisely at the end of the transition. Upon lowering the temperature further, the signal slowly decays and vanishes below 80 K, when the system transitions to the superconducting state and phase‐coherent Cooper pairs dominate the conduction.

Figure 2c shows the magnetic field dependence of PHE at 88 K in the 0–8 T field range. PHE monotonously increases with the external field strength, evidencing that it is a field‐driven effect rather than a magnetic order. By performing similar measurements at various temperatures within the superconducting transition (86–90 K), we obtain the field dependence of PHE shown in Figure 2d. The field dependence exhibits a nonlinear behavior that varies with temperature and tends to saturate at high fields. This behavior can be modeled by fitting a phenomenological equation of the form $R_{xy}\left(\theta =90^{\circ },\varphi =-45^{\circ }\right)=n_{QP}/\left(1+exp\left(H_{1}/\left(H+H_{0}\right)\right)\right)$ with the assumption that PHE may arise from spin‐polarized quasiparticles, as discussed later. In this context, $n_{QP}$ represents the saturation density of spin‐polarized quasiparticles, $H_{1}$ characterize the rate at which spin‐polarized quasiparticle density increases as the magnetic field is applied, and $H_{0}$ denotes the critical field at which the quasiparticle density begins to rise significantly. This equation provides a physically consistent framework for describing the evolution of spin‐polarized quasiparticles under an applied in‐plane magnetic field, capturing a continuous transition from the superconducting state –characterized by a negligible population of spin‐polarized quasiparticles– to the normal state, where their density increases progressively with the field strength. The temperature dependence of the fitting parameters is presented in Section S1. Figure S2 shows the behavior of $R_{xy}\left(\theta =90^{\circ },\varphi =-45^{\circ }\right)$ as a function of temperature at various magnetic fields. These measurements support our earlier observation that the PHE signal appears only in a narrow temperature range close to $T_{c}$ and its maximum value increases by increasing the magnetic field. On the contrary, the normal‐state Hall resistance is mainly field independent.

## 3 Magnetoresistance and Anomalous Hall Effects

**FIGURE 3:**
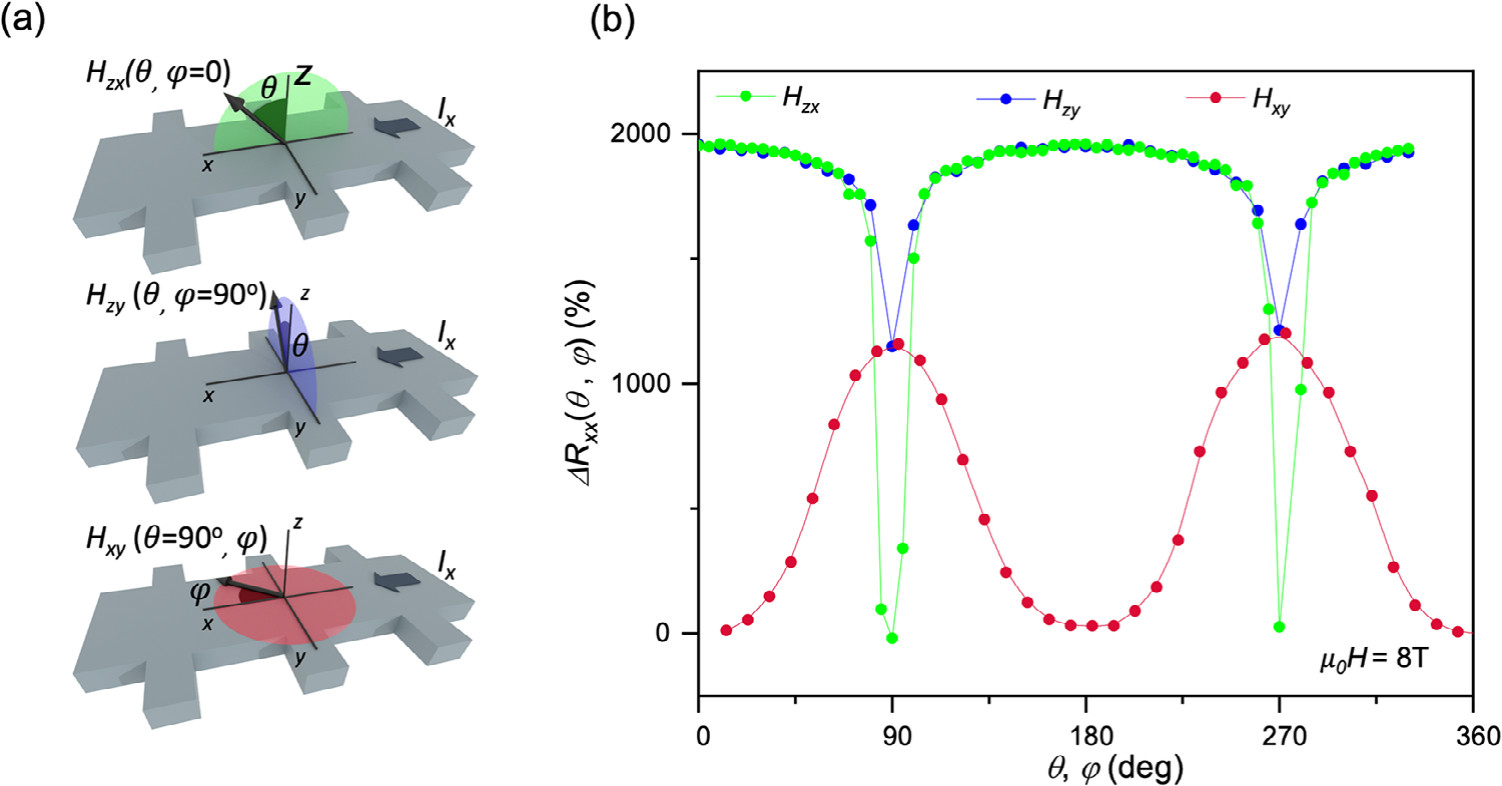
Out‐of‐plane longitudinal and Hall resistance components. (b) Angular dependence of the longitudinal component of magnetoresistance, $\Delta R_{xx}\left(\theta ,\varphi \right)$ = ($R_{xx}\left(\theta ,\varphi \right)-R_{xx}\left(\theta =0^{o},\varphi =0^{0}\right))\cdot 100/R_{xx}\left(\theta ,\varphi \right)$, obtained at 88 K and 8 T by rotating the magnetic field along the different planes illustrated in (a).

To gather further signatures of the anomalous transport behavior at the superconducting transition temperature, we measure the angular dependence of $R_{xx}$ by rotating the magnetic field in all three planes ($R_{xx}\left(\theta ,\varphi =0^{0}\right)$, $R_{xx}\left(\theta ,\varphi =90^{\circ }\right)$ and $R_{xx}\left(\theta =90^{\circ },\varphi \right)$ as schematically shown in Figure 3a at 88 K and 8 T. The results are shown in Figure 3b. The resistance is high when the magnetic field has a substantial out‐of‐plane component, i.e., far away from θ = 90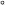 or 270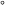. As the field approaches these angles, $R_{xx}$ undergoes sharp drops to respective resistance values for the field aligned along *x* and *y*. Notably, $R_{xx}$ does not follow a geometrical relationship with the field (i.e., simple ∝ $\cos^{2}$ θ dependence) but transits to a low‐resistance state when the in‐plane field component is significantly larger than the out‐of‐plane one, satisfied when $\theta \approx 90^{\circ }\pm$ 10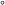 or 270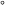 ± 10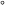. These measurements demonstrate that a strictly in‐plane field is essential for a large planar PHE, whereas an out‐of‐plane component suppresses the response and drives the film into a high‐resistance state with a weak magnetic‐field response.

**FIGURE 4:**
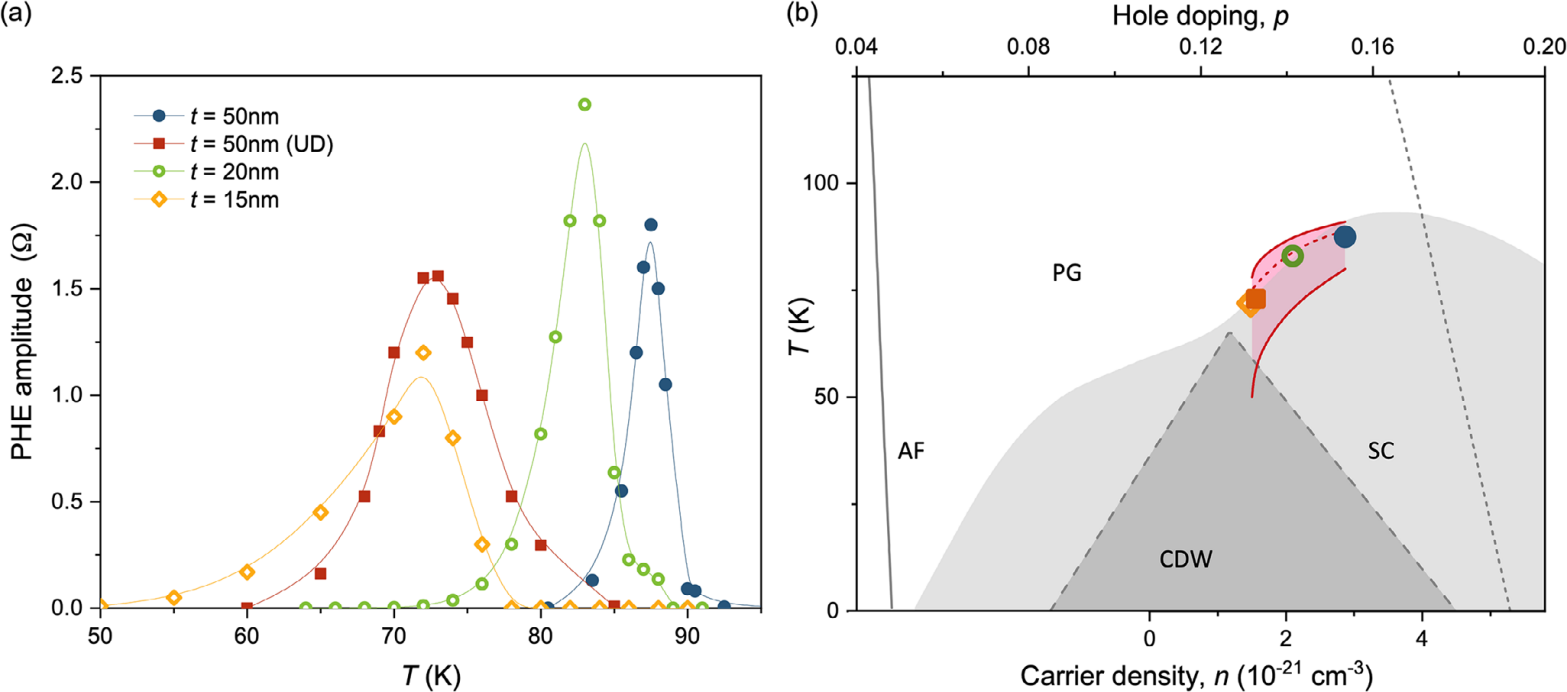
Doping‐dependent Hall measurements. (a) Temperature dependence of the PHE amplitude obtained for samples with different thicknesses and doping levels. (b) Temperature dependence of the PHE amplitude peak for the samples shown in (a). The lower x‐axis corresponds to experimental data, while the upper x‐axis is derived from the $T_{c}$ dependence. The points have been plotted in the temperature‐doping phase diagram of YBCO, highlighting the different phases appearing: antiferromagnetic (AF) order, pseudogap (PG), superconductivity (SC), and charge‐density‐wave (CDW) order [34, 35]. The red dashed area shows the temperature range in which the PHE is observed.

To assess the role of carrier doping on the observed effect, we extended the study to YBCO films of reduced thickness (15 and 20 nm), which exhibit lower $T_{c}$. While thickness reduction can introduce structural defects such as stacking faults, disorder, or strain [33], Hall measurements indicate that the dominant effect is a change in carrier density. From the linear slope of the normal‐state Hall resistance, we extract n = 1.6, 2.1, and 2.9 $\cdot 10^{-21}\mathrm{cm}$ 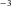 for the 15, 20, and 50 nm samples, respectively. To further isolate doping effects, we include a 50 nm film grown under reduced oxygen pressure (n= 1.5· $10^{-21}\mathrm{cm}$ 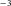; see Methods). Figure 4a shows the temperature dependence of the PHE amplitude at 8 T for different doping levels, while Figure 4b presents the temperature–carrier density phase diagram of YBCO [34, 35], with the shaded region marking the PHE regime and the largest observed values plotted as data points. In all cases, the effect is strongest near $T_{c}$ and broadens progressively as doping decreases.

Complementary Hall measurements under an out‐of‐plane magnetic field provide further evidence of anomalous transport in YBCO films (Figure S3). Here, we plot the odd component of $R_{xy}\left(\theta =0^{\circ },\varphi =0^{\circ }\right)$ to suppress contributions from vortex motion and contact misalignment (see Section S4). In the normal state (T=100K), the Hall resistance varies linearly with field, following $R_{xy}=H_{z}/\left(nqt\right)$, where q and n are the normal‐state charge and carrier densities, and t is the film thickness. Near $T_{c}$, the response becomes strongly nonlinear, exhibiting oscillations and even a sign reversal at low fields. A characteristic dip appears when the slope changes from negative (low fields) to positive (high fields), as shown in the inset of Figure S3a. Such nonlinearity is commonly associated with superconducting fluctuations and quasiparticle scattering, which depend on Fermi surface characteristics and doping [16, 19]. Figure S3b compares the temperature evolution of anomalous Hall dips (upper panel) with the PHE amplitude (lower panel) for samples spanning different doping levels, achieved through variations in thickness and oxygen content. Both features emerge near $T_{c}$ and exhibit similar trends, suggesting that they may share a common origin involving quasiparticle‐mediated processes. Magnetic field–temperature phase diagrams (see Section S5) further show that both responses display anomalies close to $T_{c}$. While AHE and PHE appear at comparable temperatures under low fields, the AHE deeps shift to lower temperatures more rapidly as the field increases, reflecting the stronger suppression of $T_{c}$ by out‐of‐plane fields. Although both effects may share a quasiparticle‐related origin, the interpretation of out‐of‐plane measurements is more complex and likely includes additional contributions. Therefore, the following discussion will focus on longitudinal and Hall transport measurements performed under an in‐plane magnetic field, which provide the most direct evidence for spin‐polarized quasiparticle dynamics.

## 4 Signatures of the Spin–Orbit Coupling

Linear magnetotransport measurements in YBCO thin films reveal a pronounced in–plane ADMR and PHE. Both responses are observed only within a narrow temperature interval below $T_{c}$, are induced by the applied magnetic field, and vanish at zero applied field. The angular dependence of the signal is governed by the relative orientation between the magnetic field and the current direction, with no detectable dependence on the crystallographic axes (see Section S6), indicating that the effect is not tied to lattice symmetry.

AMR and PHE are typically observed in magnetic materials and in certain non‐magnetic quantum materials with topological properties [24, 25]. In non‐magnetic systems, the PHE has been associated with a chiral‐anomaly related transport in topological states, often linked to spin‐textured bands and non‐trivial Berry curvature [36]. Large AMR and PHE signals have also been reported in topological superconductors exhibiting strong SOC [37, 38, 39, 40]. In these systems, however, the anisotropic response typically persists over a wide temperature range extending well above the superconducting transition and is commonly attributed to topological surface or bulk states, which provide an experimental manifestation of non–trivial Berry curvature and chiral–anomaly physics. In cuprates, in‐plane transport anisotropy has previously been reported in single crystals with broken rotational symmetry [21, 22, 23], where it was attributed to electronic nematicity, i.e., a spontaneous breaking of rotational symmetry, which can occur even in the absence of an external magnetic field. This scenario is incompatible with the present observations, as the PHE signal is strictly field–induced and vanishes at zero field. Anisotropic in‐plane magnetoresistance has also been reported in [41], where it was attributed to spin‐dependent scattering arising from antiferromagnetic order in the ${\mathrm{CuO}}_{2}$ planes. Such mechanisms typically give rise to angular responses that depend on the orientation of the magnetic field relative to the crystallographic axes. By contrast, the absence of any crystallographic anisotropy in the angular dependence of the magnetoresistance rules out static magnetic order or lattice–symmetry–driven mechanisms as the dominant origin of the observed linear response. The behavior observed in the present cuprate films is therefore qualitatively distinct from these previously reported scenarios.

### 4.1 Cooperative Quasiparticle‐Driven Transport Scenario

Motivated by these experimental constraints, we consider a cooperative mechanism in which several effects become simultaneously relevant with the onset of superconducting coherence.

Recent spin‐ and angle‐resolved photoemission spectroscopy (SARPES) studies on bismuth‐based cuprates have revealed unexpected spin textures in the quasiparticle states, including nonzero spin polarization reaching up to 40 % in certain regions, spin–momentum locking, persisting in both the normal and superconducting states [26, 28]. These observations have been attributed to an intrinsic Rashba–type spin–orbit coupling (SOC) arising from local inversion symmetry breaking associated with the layered crystal structure of cuprates and local structural distortions. Specifically, the asymmetric environment around Cu atoms in the ${\mathrm{CuO}}_{2}$ planes, combined with Jahn–Teller distortions, oxygen modulations, and buckling of the ${\mathrm{CuO}}_{2}$ layers can generate internal electric fields within the unit cell that facilitate SOC and spin–momentum locking. The confinement of the observed magnetotransport response to a narrow temperature window near $T_{c}$ does not imply the absence of SOC at higher temperatures, but rather reflects the fact that SOC alone is generally insufficient to produce measurable transport signatures in the normal state. The superconducting transition may therefore be viewed as enhancing the sensitivity of transport to these pre–existing SOC effects, through a combination of field–induced quasiparticle asymmetry and the emergence of coherent quasiparticle dynamics, as discussed below.

(i) Zeeman‐induced spin polarization of superconducting quasiparticles: In the superconducting state, Zeeman splitting of the density of states (DOS) introduces a spin‐dependent particle–hole asymmetry of Bogoliubov quasiparticles. This asymmetry can give rise to spin‐polarized quasiparticles and an effective spin–charge decoupling, even in non‐magnetic systems under in‐plane magnetic fields [10, 14, 15]. Because this mechanism relies on the unique particle‐hole mixing intrinsic to the superconducting state, it becomes operative only below the onset of superconductivity. This behavior is therefore consistent with the observation that the onset of the PHE coincides with the opening of the superconducting gap.
(ii) Coherence‐enhanced quasiparticle transport near $T_{c}$: The confinement of the observed PHE to a narrow temperature range near $T_{c}$ is consistent with a quasiparticle–mediated transport mechanism that is enhanced by the onset of superconducting coherence. Time‐resolved optical studies in cuprate superconductors have shown that the quasiparticle recombination lifetime exhibits a local maximum near $T_{c}$ [42], reflecting a transition from a phase‐fluctuating state to a phase‐coherent condensate. As quasiparticle dynamics is strongly influenced by the emergence of coherence, quasiparticle–driven transport processes are expected to be maximized in the vicinity of the transition [13]. The peak observed in the PHE signal is therefore consistent with a local enhancement of quasiparticle lifetime and coherence near $T_{c}$.
(iii ) SOC‐driven spin–momentum locking: The SOC landscape, arising from local inversion symmetry breaking, appears to be a generic property of the ${\mathrm{CuO}}_{2}$ planes [26, 28]. Its intrinsic nature is supported by experimental observations showing that suppressing structural distortions reduces the measured spin polarization [28]. In this framework, the SOC strength is estimated to be at least comparable to the interlayer hopping energy ($t_{⊥}∼$ 20 meV), which, although smaller than typical values in topological insulators or heavy metals, is on the order of the superconducting gap and thus significant for spin transport. In [29] the SOC is modeled as a dimensionless parameter, λ, scaled by the nearestneighbour hopping energy $t_{1}$. For typical cuprate values of $t_{1}$ ≈ 0.3–0.4 eV for cuprates [43], their simulations using λ = 0.08 $t_{1}$ (24–32 meV) reproduce spintronic phenomena such as the spin Hall and Edelstein effects within a coherent band–structure framework. The emergence of long–lived quasiparticles in the superconducting state of cuprates provides conditions under which such SOC–driven transport responses can become experimentally observable.

In summary, while SOC is expected to persist over a broad temperature range, the emergence of superconducting coherence and Zeeman–induced quasiparticle asymmetry near $T_{c}$ provides a cooperative framework in which SOC–related transport signatures become experimentally accessible.

Within this framework, the intrinsic nanoscale inhomogeneity of cuprate superconductors provides a natural microscopic environment for local inversion symmetry breaking. Cuprates are widely recognized as intrinsically nanoscale inhomogeneous materials, in which spin, charge, and lattice degrees of freedom are strongly entangled at short length scales. Experimental evidence indicates that superconductivity in these systems coexists with electronic and structural inhomogeneity, rather than forming a uniform long–range phase. Charge‐density‐wave (CDW) order and lattice modulations self–organize into nanoscale striped or puddle–like domains, reflecting locally ordered lattice distortions embedded in a quenched disordered background [44, 45, 46, 47, 48]. This intrinsic heterogeneity is closely linked to the formation and coexistence of interacting defect networks, including ordered oxygen interstitials, ${\mathrm{CuO}}_{2}$ plane buckling, and incommensurate local lattice distortions whose spatial organization correlates with the superconducting critical temperature, establishing “optimum inhomogeneity” as a fundamental materials parameter [45]. Such nanoscale structural complexity has been shown to strongly influence key electronic properties, including the Fermi–surface reconstruction, the pseudogap energy scale, spin– and CDW correlations, and ultimately the superconducting critical temperature. Although the emergence of these striped and puddled domains is an intrinsic property of the cuprates, their microscopic arrangement can be tuned through external parameters such as local thermal treatments, providing a route to control superconductivity via manipulation of the underlying nanoscale phase separation and defect landscape [47]. At a more general level, the coexistence of CDW puddles with correlated disorder gives rise to a highly non–uniform electronic and structural landscape, accompanied by dynamic local lattice distortions that lower crystal symmetry on nanometre length scales [49, 50]. Taken together, these observations provide a consistent microscopic basis for locally broken inversion symmetry in cuprate superconductors, which can give rise to spin–orbit coupling effects, even when the overall crystallographic structure remains centrosymmetric.

It is important to note that structural transitions and lattice fluctuations can differ substantially between polycristalline, single crystals, and samples with varying surface‐to‐volume ratios [51]. Thus, the degree of local symmetry breaking and the associated spin–orbit coupling effects may be strongly influenced by factors such as film thickness, strain, substrate, growth conditions, and defects introduced during device fabrication. These considerations highlight the practical challenges of controlling disorder in cuprate‐based devices, which require a well‐optimized and reproducible fabrication process.

### 4.2 Nonlinear Magnetotransport

**FIGURE 5:**
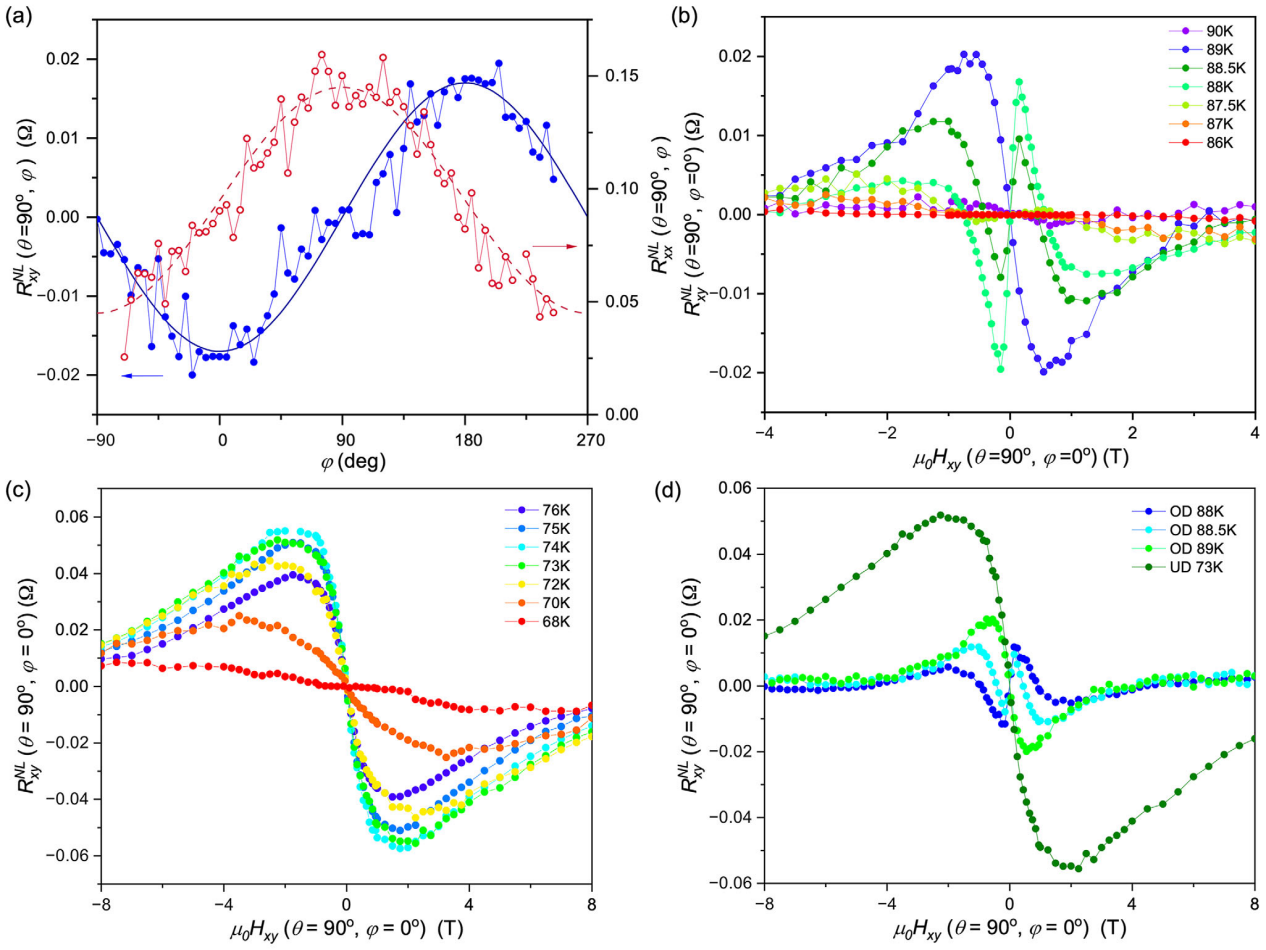
Nonlinear transverse and longitudinal magnetoresistance components. (a) In‐plane angular dependence of the nonlinear Hall resistance (closed symbols, left axis) and magnetoresistance (open symbols, right axis) measured at 89 K and 0.35 T. Solid and dashed lines show the angular dependence of cos(φ) and sin(φ), respectively. (b,c) Magnetic field dependence of the nonlinear PHE measured at φ = 0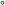 and at various temperatures for a 50 nm YBCO optimally doped and underdoped devices, respectively. (d) Comparison of the magnetic field dependence of the nonlinear transverse resistance measured at 88, 88.5 and 89 K for a 50 nm optimally doped device (OD) and 73 K for a 50 nm underdoped device (UD).

Additional insight is obtained from nonlinear magnetotransport measurements. In systems with topologically protected spin‐polarized transport, longitudinal and transverse resistances also entail nonlinear components [52, 53, 54, 55]. To investigate this aspect, we characterize the nonlinear response of YBCO by extending the analysis to the second order in the applied current, such that $V_{xy}$ = $R_{xy}I_{x}$ + $R_{xy}^{NL}I_{x}^{2}$ and $V_{xx}$ = $R_{xx}I_{x}$ + $R_{xx}^{NL}I_{x}^{2}$. $R_{xy}^{NL}$ and $R_{xx}^{NL}$ have then been determined by taking the difference of transverse and longitudinal components of magnetoresistance under positive, $I_{x}^{+}$, and negative, $I_{x}^{-}$, currents. Figure 5a shows the angular dependence of the nonlinear transversal and longitudinal components obtained at 89 K, 0.35 T. $R_{xy}^{NL}$ and $R_{xx}^{NL}$ exhibit cos(φ) and sin(φ) dependences, respectively, with a periodicity of 2π, consistent with observations in non‐magnetic topological insulators [52, 53, 55]. Figure 5b,c displays $R_{xy}^{NL}$ measured at different temperatures across the transition for a 50 nm‐thick optimally doped and underdoped YBCO film, respectively, as a function of magnetic field applied parallel to the current ($\theta =90^{\circ },\varphi =0^{\circ }$). In both samples, a clear nonlinear response develops within the same narrow temperature range where the linear PHE is observed.

In the optimally doped sample, $R_{xy}^{NL}$ becomes detectable near 89 K, increases with magnetic field up to 0.6 T, and it is progressively suppressed at higher fields, vanishing at 4 T. Upon further cooling, a reversal of the sign of $R_{xy}^{NL}$ is observed at 88.5 K, indicating the presence of competing contributions to the nonlinear response. By contrast, the underdoped sample exhibits a substantially larger nonlinear signal that persists to higher magnetic fields while maintaining the same sign over the measured temperature range. Figure 5d summarizes the combined influence of temperature and doping on the nonlinear response, highlighting both the enhanced magnitude of $R_{xy}^{NL}$ in the underdoped sample and the sign reversal obtained for the optimally doped sample. Given that charge–density–wave correlations are stronger in underdoped cuprates, this behavior is consistent with an enhanced effectiveness of SOC–mediated transport processes in the presence of CDW–related electronic and structural inhomogeneity discussed above. While further investigation is required to clarify the microscopic mechanism, this trend supports the view that CDW correlations can amplify the observed nonlinear response. Additional $R_{xy}^{NL}$ data and analysis are presented in Section S7, where the field dependence is evaluated at different in‐plane magnetic field orientations, consistently showing the expected cos(φ) dependence. Independent measurements of the second‐harmonic response, shown in Section S8, are in good agreement with the nonlinear resistance extracted using the current‐polarity method, confirming the internal consistency of the two approaches.

**FIGURE 6:**
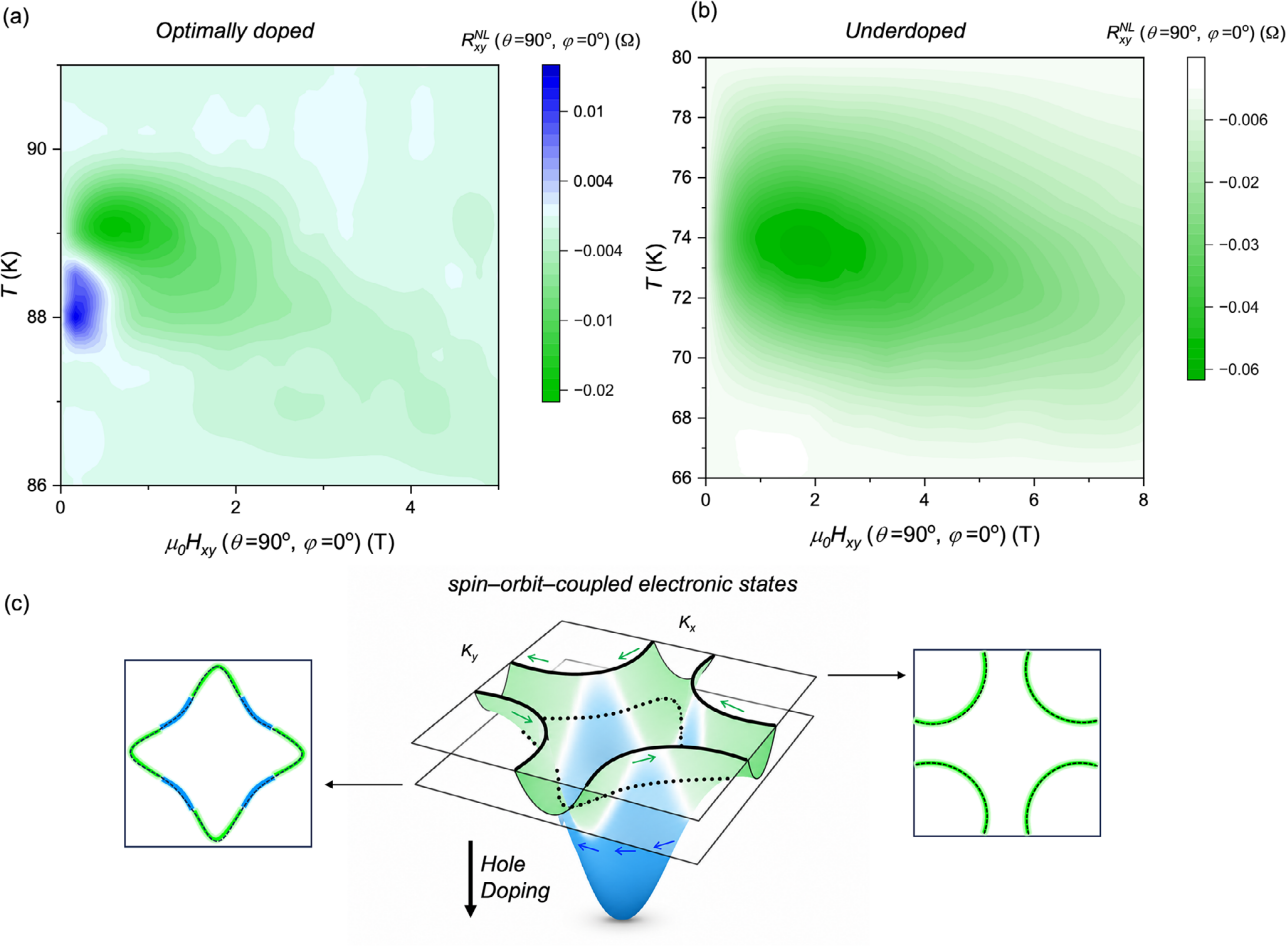
Temperature–magnetic–field evolution of the nonlinear transverse resistance and schematic spin–orbit‐coupled electronic landscape. (a,b) Magnetic‐field‐temperature maps of the nonlinear transverse resistance for optimally doped and underdoped YBCO thin films, respectively. (c) Schematic representation of SOC electronic structure inspired by spin–resolved ARPES measurements on Bi–based cuprates [28]. The color coding illustrates opposite spin helicities (green: counter‐clockwise; blue: clockwise) observed in those systems. The left and right panels schematically depict how changes in hole doping can modify the dominant quasiparticle states sampled near the Fermi level. This schematic is intended as a qualitative framework and does not represent a direct measurement of the YBCO electronic structure.

Figure 6 presents magnetic‐field‐temperature maps of $R_{xy}^{NL}$ for the optimally doped and underdoped YBCO films, providing a compact visual summary of the trends discussed above. In the optimally doped sample (Figure 6a), the nonlinear response is confined to a narrow temperature window near $T_{c}$ and exhibits a clear sign reversal as a function of temperature and magnetic field, consistent with the behavior shown in Figure 5b. In contrast, the underdoped sample (Figure 6b) displays a larger nonlinear signal that persist over a broader field and temperature range while preserving the same sign, in agreement with the data presented in Figure 5c.

The two panels of Figure 6a,b can be placed in qualitative correspondence with the evolution of spin–polarized quasiparticle states reported by spin–resolved ARPES studies on Bi–based cuprates [28]. In particular, they report that the spin texture of low‐energy quasiparticle states in those materials evolves systematically with doping and binding energy, including changes in the spin helicity. Within a phenomenological framework, the temperature‐driven sign reversal of $R_{xy}^{NL}$ in the optimally doped film and its absence in the underdoped film are consistent with a change in the dominant quasiparticle contribution to transport as a function of doping and energy scale. The schematic representation of Figure 6c illustrates this correspondence at the conceptual level. The diagram depicts a generic spin–orbit‐coupled electronic structure inspired by SARPES results [28]. In this picture, the nonlinear transport does not probe the spin texture directly but reflects the cumulative contribution of quasiparticles with different spin–momentum characteristics to the transport response. The enhanced and sign–preserving nonlinear signal observed in the underdoped regime is thus consistent with a more robust contribution from a single dominant quasiparticle population, whereas the sign reversal in the optimally doped sample suggests competition between quasiparticles with different effective spin character.

Within the framework described above, the nonlinear resistance components, $R_{xy}^{NL}$ and $R_{xx}^{NL}$ can be interpreted in terms of spin‐polarized quasiparticles interacting with an induced in‐plane field, orthogonal to both the current direction and the z‐axis, that may arise from Rashba‐type SOC. We propose that the scattering rate of spin‐polarized quasiparticles could differ depending on whether their spin orientation is parallel or antiparallel to the intrinsic Rashba field, assumed to lie along the y‐axis. This asymmetry might give rise to a bilinear (unidirectional) magnetoresistance that depends on the current direction or field reversal and scales linearly with the injected current [52, 56]. Analogous to the relationship between ADMR and PHE, a similar effect is also expected in $R_{xy}^{NL}$ with a 90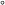 phase shift, as shown in Figure 5a.

The initial increase of the signal with magnetic field is consistent with the field‐induced generation of spin‐polarized quasiparticles near $T_{c}$, which is necessary to generate the magnetoresistance asymmetry. The subsequent decay with increasing field strength suggests a complex interplay between external and intrinsic fields acting on the quasiparticles. The observed dependence of the sign and magnitude of $R_{xy}^{NL}$ on temperature and doping is therefore compatible with a quasiparticle‐mediated mechanisms operating within a SOC electronic landscape. We emphasize that this interpretation is intentionally qualitative; while it captures the main experimental trends and their systematic evolution and doping, further experimental and theoretical work will be required to establish a quantitative microscopic description of the nonlinear response and its relation to the detailed spin texture of the quasiparticle states.

Linear and nonlinear magnetoresistive responses have attracted renewed attention in condensed–matter physics, as they provide sensitive probes of the electronic structure at the Fermi level, spin–orbit coupling, spin–momentum locking, and related geometric properties of electronic bands in a wide range of materials [52, 55, 57, 58, 59, 60]. Extending such studies to high–temperature superconductors, which host a particularly rich and strongly correlated electronic landscape, offers a complementary route to accessing quasiparticle dynamics beyond the conventional normal–state transport regime. In this context, the present measurements provide a means to probe quasiparticles in regions of the cuprate phase diagram where superconductivity coexists with CDW order (see Figure 4b). Quasiparticles excited from the superconducting phase are phase‐coherent linear superpositions of normal‐state electrons and holes, while charge–density wave quasiparticles consist of superpositions of electrons (or holes). As a consequence, the recombination dynamics of these excitations–particularly their temperature and magnetic field dependence–are expected to be sensitive to carrier doping [42]. The systematic evolution of the nonlinear transport response with doping observed here is therefore consistent with a modulation of quasiparticle behavior across the phase diagram. Although the phase diagram shown refers to bulk YBCO, the underlying physics is expected to be intrinsic to the ${\mathrm{CuO}}_{2}$ planes. In thin–film systems, structural and electronic responses may be modified by epitaxial strain, reduced dimensionality, and disorder, which can influence the magnitude and experimental visibility of the observed effects without altering their intrinsic origin. Within these constraints, the nonlinear magnetotransport signatures reported here highlight the potential of current–dependent transport measurements as a tool for investigating spin–orbit–coupled quasiparticle dynamics in high–temperature superconductors.

## 5 Summary and Outlook

High‐temperature cuprate superconductors have traditionally been regarded as systems dominated by strong electronic correlations with relatively weak SOC. However, recent reports of spin–momentum locking in bismuth‐based cuprates suggest that the spin landscape in these materials may be more complex than previously assumed. Despite these developments, the microscopic origins, intrinsic characteristics, and broader implications of SOC in cuprates remain incompletely understood. In this study, we present magnetoresistance and Hall effect measurements near the superconducting transition in YBCO that are consistent with the presence of spin‐dependent transport phenomena. These observations may reflect the influence of Rashba‐type SOC on spin‐polarized quasiparticle dynamics. While this interpretation challenges the conventional view of SOC negligible role in cuprates, we emphasise that further investigation is required to establish its significance and underlying mechanisms. The interplay between SOC, strong correlations, and superconductivity represents a promising avenue for future research in condensed matter physics. Continued development of experimental techniques to probe these interactions could yield deeper insights into the electronic structure and transport properties of high‐temperature superconductors. In particular, understanding how lattice symmetry and doping modulate these effects may contribute to a more comprehensive picture of the cuprate phase diagram. Beyond fundamental interest, the possibility of spin–orbit‐driven effects in high‐temperature superconductors raises intriguing prospects for spintronic applications operating at liquid nitrogen temperatures. However, realising such applications will depend on a clearer understanding of the relevant mechanisms and their reproducibility across different material systems.

## 6 Methods

### 6.1 Sample Growth

Epitaxial ${\mathrm{YBa}}_{2}{\mathrm{Cu}}_{3}O_{7-x}$ films of thicknesses 50, 20, and 15 nm were grown on top of ${\mathrm{LaAlO}}_{3}$ (LAO), ${\mathrm{SrTiO}}_{3}$ (STO) and ${\left({\mathrm{LaAlO}}_{3}\right)}_{0.3}-{\left({\mathrm{Sr}}_{2}{\mathrm{AlTaO}}_{6}\right)}_{0.7}$ (LSAT) single crystal substrates by pulsed laser deposition. Substrates were heated up to 810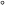 C, with an oxygen partial pressure of 0.3 mbar, with a target‐to‐substrate distance of 52.5 mm. Ablation of the target material was achieved by using a high fluence laser operating at 2 J/${\mathrm{cm}}^{2}$ and a frequency of 5 Hz. During cooling to room temperature, the samples were kept in an oxygen atmosphere of 1 Bar to ensure proper oxygenation of the deposited thin films. An under‐doped 50 nm film was grown by post‐annealing for 3 h at 450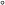 C in a nitrogen atmosphere. Microstructural characterization of YBCO thin films grown in similar conditions was reported elsewhere [61].

### 6.2 Device Fabrication and Measurements

Four‐terminal Hall devices were fabricated using standard photolithography and wet etching techniques with dimensions of 20–30 μm in length and 20–50 μm in width. Au contact electrodes with a thickness of 50 nm were deposited by sputtering. The contact resistance at 80 K was $∼5\cdot 10^{3}$ $\Omega \cdot cm^{2}$. Transverse and longitudinal magnetoresistance components were measured down to 10 K, using a Physical Property Measurement System (PPMS, Quantum Design) equipped with a sample rotator. The magnetic field was applied at various orientations by adjusting the sample's position relative to the field. A d.c. current of 5 mA was applied along the longitudinal direction. The critical temperature was determined from the maximum of the first derivative of $R_{xx}\left(T\right)$. The carrier density was calculated as $n=1/(qR_{H})$, where $R_{H}=t\left(dR_{\mathrm{xy}}/dH_{z}\right)$ is the Hall coefficient t the sample thickness and $dR_{\mathrm{xy}}/dH_{z}$ the linear slope of the Hall resistance with the out‐of‐plane magnetic field, measured by applying a ramp of –5 to 5 T.

## Conflicts of Interest

The authors declare no conflicts of interest.

## Supporting information


**Supporting File**: advs76166‐sup‐0001‐SuppMat.pdf.

## Data Availability

The data that support the findings of this study are available from the corresponding author upon reasonable request.
