# Supplementary material for: Magnetotransport Signatures of Spin–Orbit Coupling in High‐Temperature Cuprate Superconductors
Source: Adv Sci (Weinh). 2026 Jul 17;13(52):e76166. doi: 10.1002/advs.76166 (PMC13377749; doi:10.1002/advs.76166)
Supplement: Supplementary file 1 [file ADVS-13-e76166-s001.pdf]

Supplementary Information for

Magnetotransport signatures of spin-orbit coupling in high-temperature cuprate superconductors

Aleix Barrera[1], Huidong Li[1], Thomas Gunkel[1], Jordi Alcalà[1], Silvia Damerio[1], Can Onur Avci[1], Anna Palau[1]

Institut de Ciència de Materials de Barcelona, ICMAB-CSIC, Campus UAB, Bellaterra, Barcelona, 08193, Spain

List of Contents

S1. Fittings of the PHE magnetic field dependence at different temperatures.

S2: Temperature and Magnetic field dependence of $R_{xy}(\theta = 90^{o}, \varphi = -45^{o})$.

S3. Anomalous Hall Effect deep in YBCO films of different thicknesses

S4. Longitudinal and Hall magnetoresistance components under out-of-plane magnetic field.

S5. Magnetic field-temperature AHE and PHE diagrams.

S6. Devices patterned with different in-plane crystallographic orientations.

S7. Magnetic field dependence of nonlinear transverse resistance at different orientations.

S8. Validation of Nonlinear Magnetotransport via Second Harmonic measurements

S1. Fittings of the PHE magnetic field dependence at different temperatures

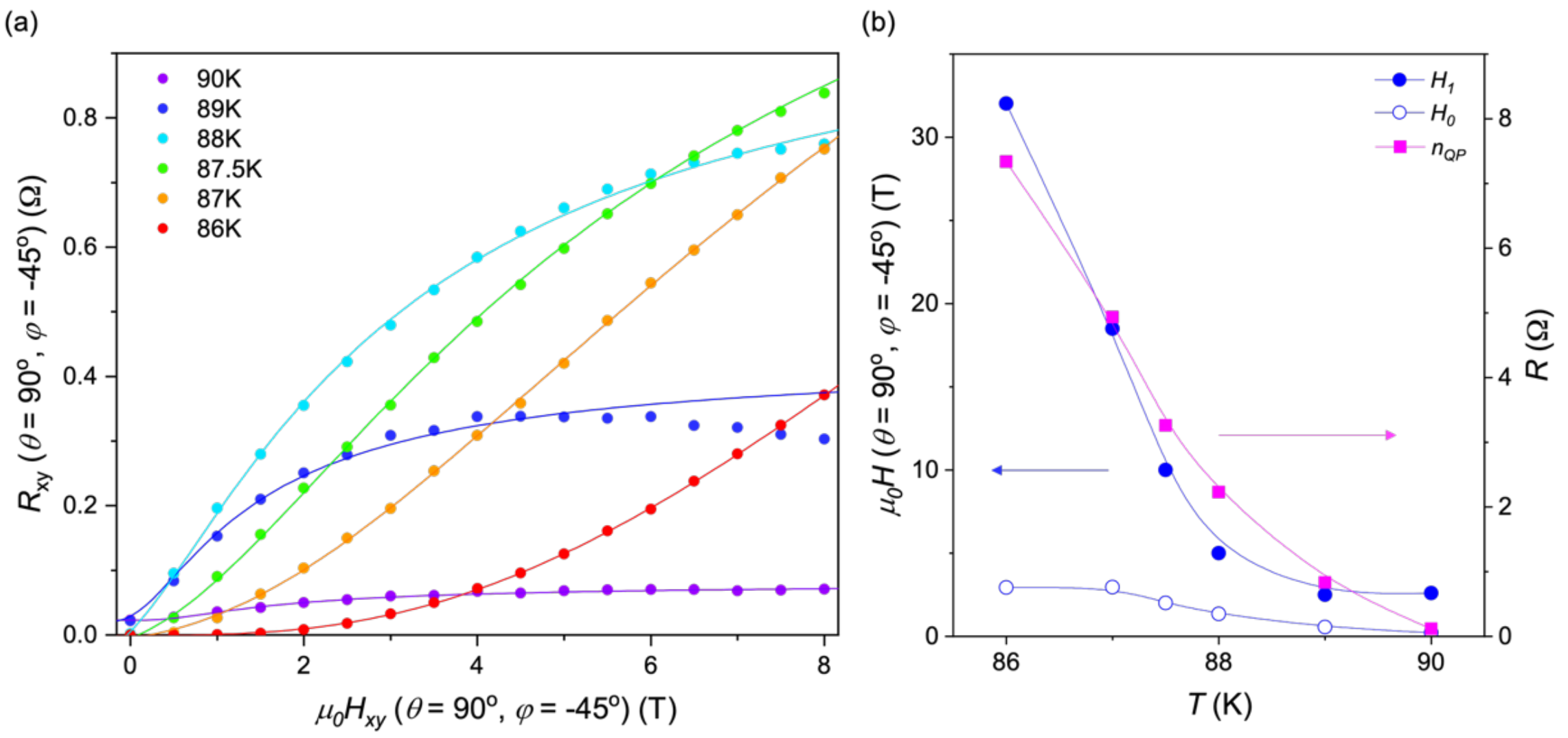

Figure S1- a) Magnetic field dependence of R_{xy} at φ = -45°. Solid lines are fits to the equation [1]. b) Temperature dependence of the fitting parameters.

Figure S1a illustrates the magnetic field dependence of the planar Hall effect (PHE) variation at several temperatures. The data are well described by the following phenomenological expression:

$$R_{xy} = \frac{n_{QP}}{1+\exp\left(H_1/(H+H_0)\right)} \qquad [1]$$

Here, n_{QP} represents the saturation density of spin-polarised quasiparticles. The parameter H_1 characterises the rate at which n_{QP} increases with the in-plane magnetic field, with smaller values indicating a steeper increase. H_0 denotes the magnetic field value at which the quasiparticle density begins to be appreciable. Figure S1b depicts the temperature dependence of the fitting parameters.

The observed increase in H_0 and H_1 with decreasing temperature is consistent with the expectation that stronger magnetic fields are required to break Cooper pairs at lower temperatures, where thermal excitation is reduced. The enhancement of n_{QP} at lower temperatures can be understood by considering that, although the energy required to break Cooper pairs increases as the temperature decreases, the application of a sufficiently strong magnetic field can still induce pair breaking. Under these conditions, the resulting spin-polarised quasiparticles are more likely to be stable at lower temperatures, leading to an increased density as the temperature is reduced. While this interpretation remains qualitative, the fitting provides a useful framework for comparing the field response across different temperatures.

S2. Temperature and Magnetic field dependence of $R_{xy}(\theta = 90^{o}, \varphi = -45^{o})$.

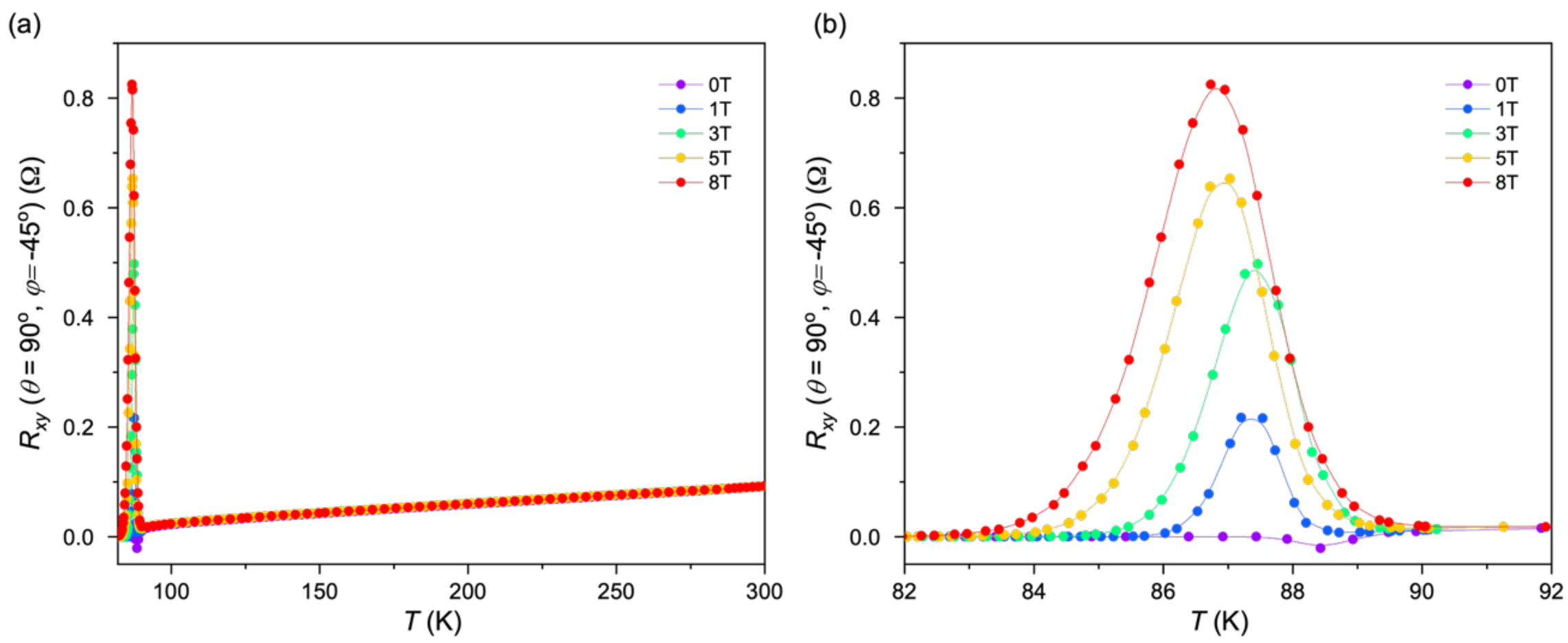

Figure S2- a) Temperature dependence of the Hall resistance measured under several in-plane magnetic fields applied at an angle of φ = -45°, corresponding to a maximum of the PHE signal. b) Magnified view of the curves in panel a), highlighting the behaviour near the superconducting transition temperature.

S3. Anomalous Hall Effect deep in YBCO films of different thicknesses

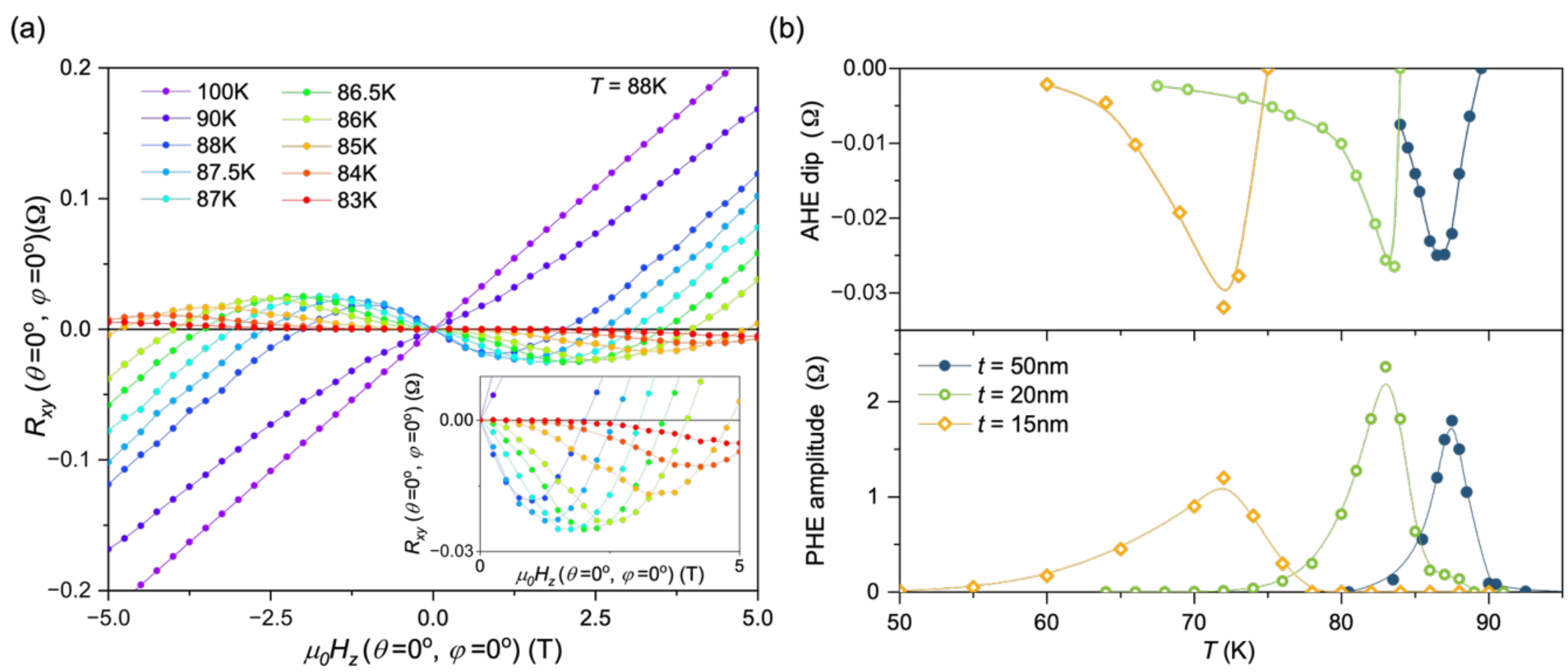

Figure S3- a) Out-of-plane magnetic field dependence of the odd component of the Hall magnetoresistance, R_{xy}, measured at different temperatures for a typical YBCO device of 50nm. The inset shows a zoom of the curves presented in the main panel. b) Temperature dependence of the AHE dip and the PHE amplitude obtained for samples with different thicknesses.

S4. Longitudinal and Hall magnetoresistance components under out-of-plane magnetic field

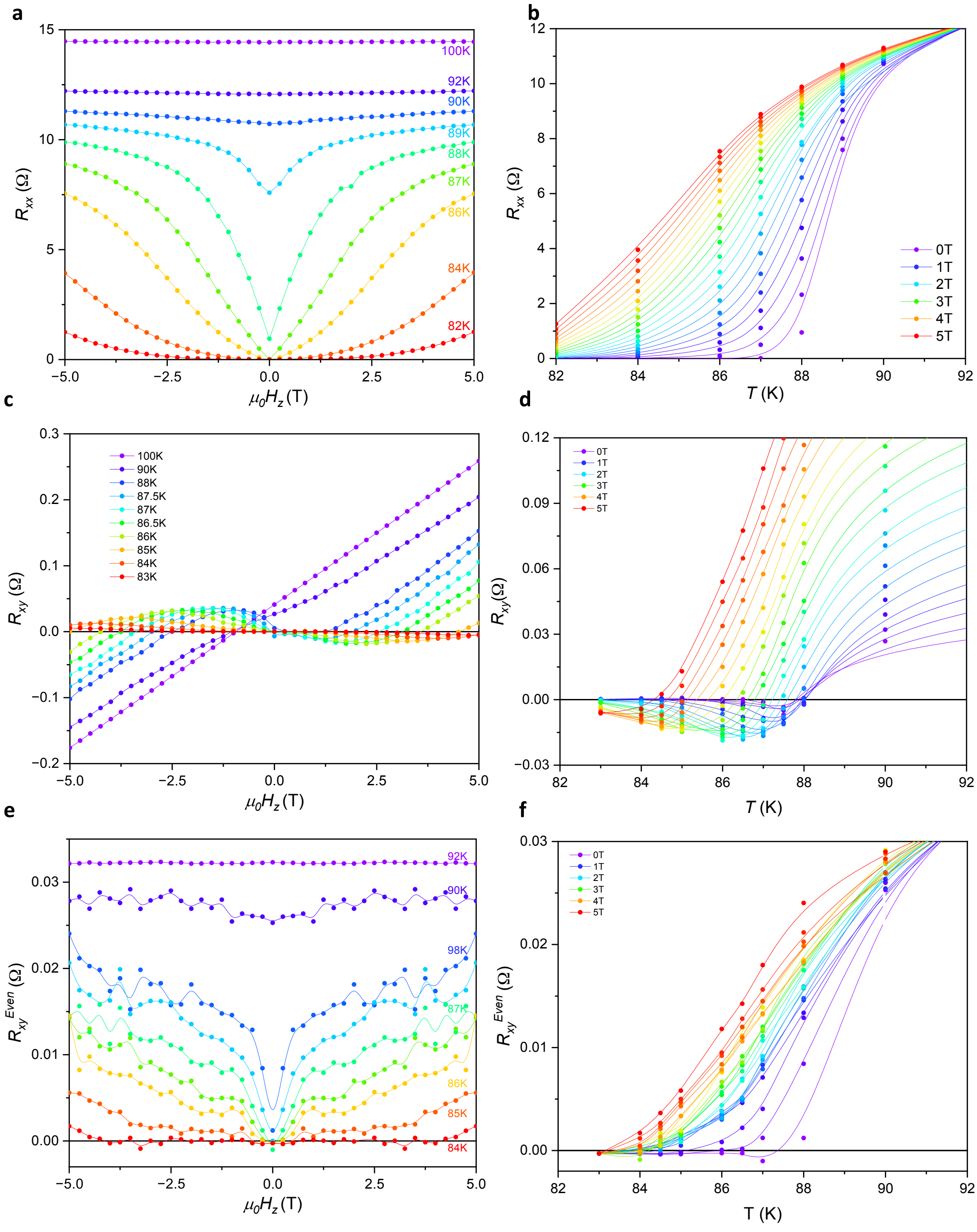

Figure S4- a,b), Magnetic field and temperature dependence of the longitudinal magnetoresistance component measured with out-of-plane magnetic field (θ=0º, φ=0º) for a YBCO device of 50nm. c, d) Magnetic field and temperature dependence of the Hall magnetoresistance measured in the same magnetic field configuration for the same device. e, f) Even component of the Hall magnetoresistance determined from the data shown in c,d.

Figure S4- a,b show the magnetic field and temperature dependencies of the longitudinal magnetoresistance component, measured with an out-of-plane magnetic field configuration, $R_{xx}(\theta=0^{o}, \varphi=0^{o})$, across the temperature-field region across the superconducting transition. The observed behavior is consistent with the expected magnetoresistance resulting from vortex motion in the mixed state.

Figure S4c,d shows the transverse component of the magnetoresistance, R_{xy}, in the same temperature-field region. To better analyse the different effects that can contribute to the out-of-plane R_{xy} signal, we calculated the even and odd components with equations [2] and [3], respectively.

$$R_{xy}^{even} = \frac{R_{xy}(H_z)+R_{xy}(-H_z)}{2} \qquad [2]$$

$$R_{xy}^{odd} = \frac{R_{xy}(H_z)-R_{xy}(-H_z)}{2} \qquad [3]$$

The Hall signal related to the odd component is responsible for producing nearly all of the Hall signal, and it shown in Figure S3 and thoroughly discussed in the main text. In contrast, the even component, shown in Figure S4e,f, contributes with a minor signal that closely resembles the one obtained for R_{xx} (Figure S4a,b), which may be attributed to contact misalignment. Additionally, anomalous Hall effects arising from vortex motion, $R_{xy,V}$ may also play a role in the even contribution [Wördenwever et al. Physica C 434, 101 (2006)]. Here, $R_{xy,V} = v_x H_z w$, where v_x is the vortex velocity along the current direction, H_z, the out-of-plane magnetic field, and w is the sample width.

S5. Magnetic field-temperature AHE and PHE diagrams.

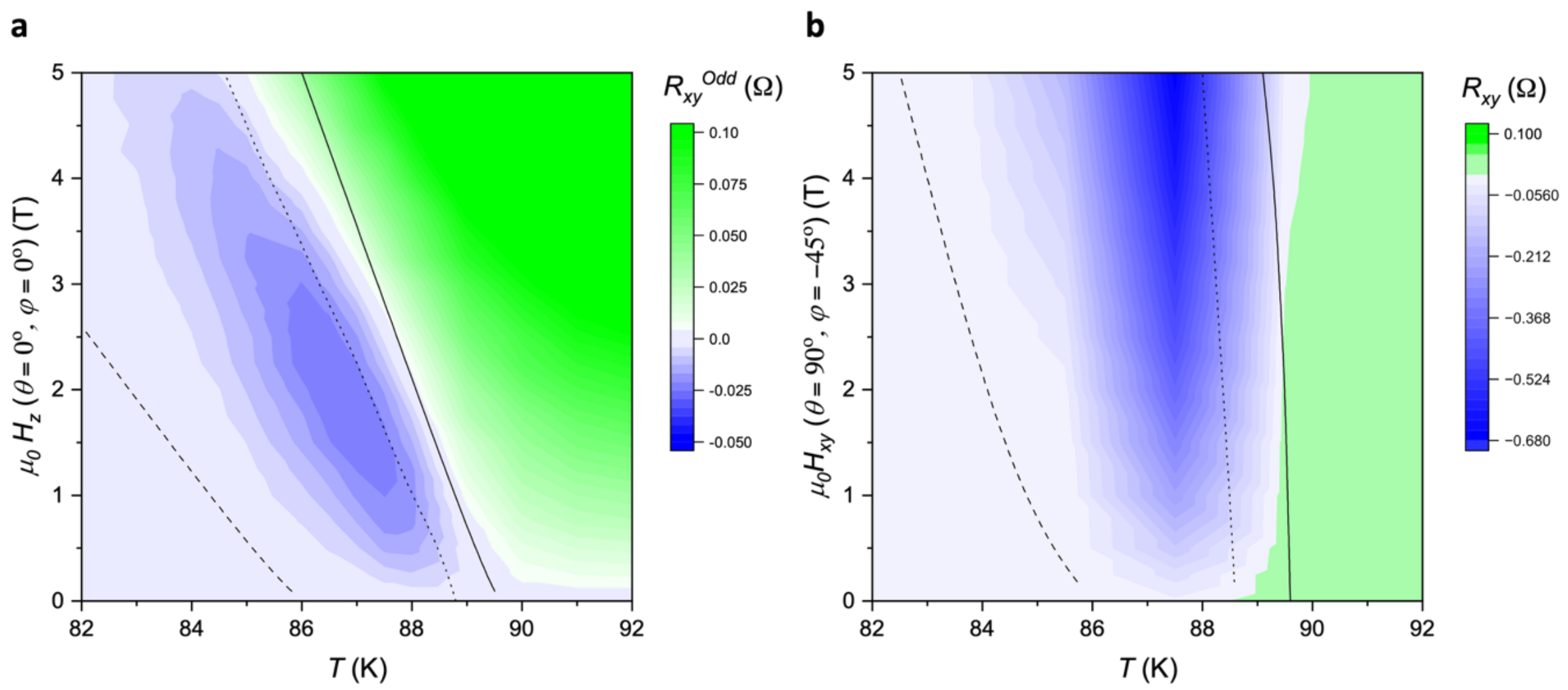

Figure S5- a) 2D diagram of the out-of-plane magnetic field-temperature dependence of the odd component of the Hall magnetoresistance and b) the in-plane magnetic field-temperature dependence of the Hall magnetoresistance at φ = -45 °. Negative values for the AHE and PHE are shown in blue. Solid, dotted, and dashed lines represent the T_c onset, middle, and zero values, respectively, calculated from out-of-plane and in-plane $R_{xx}(T,H)$.

S6. Devices patterned with different in-plane crystallographic orientations.

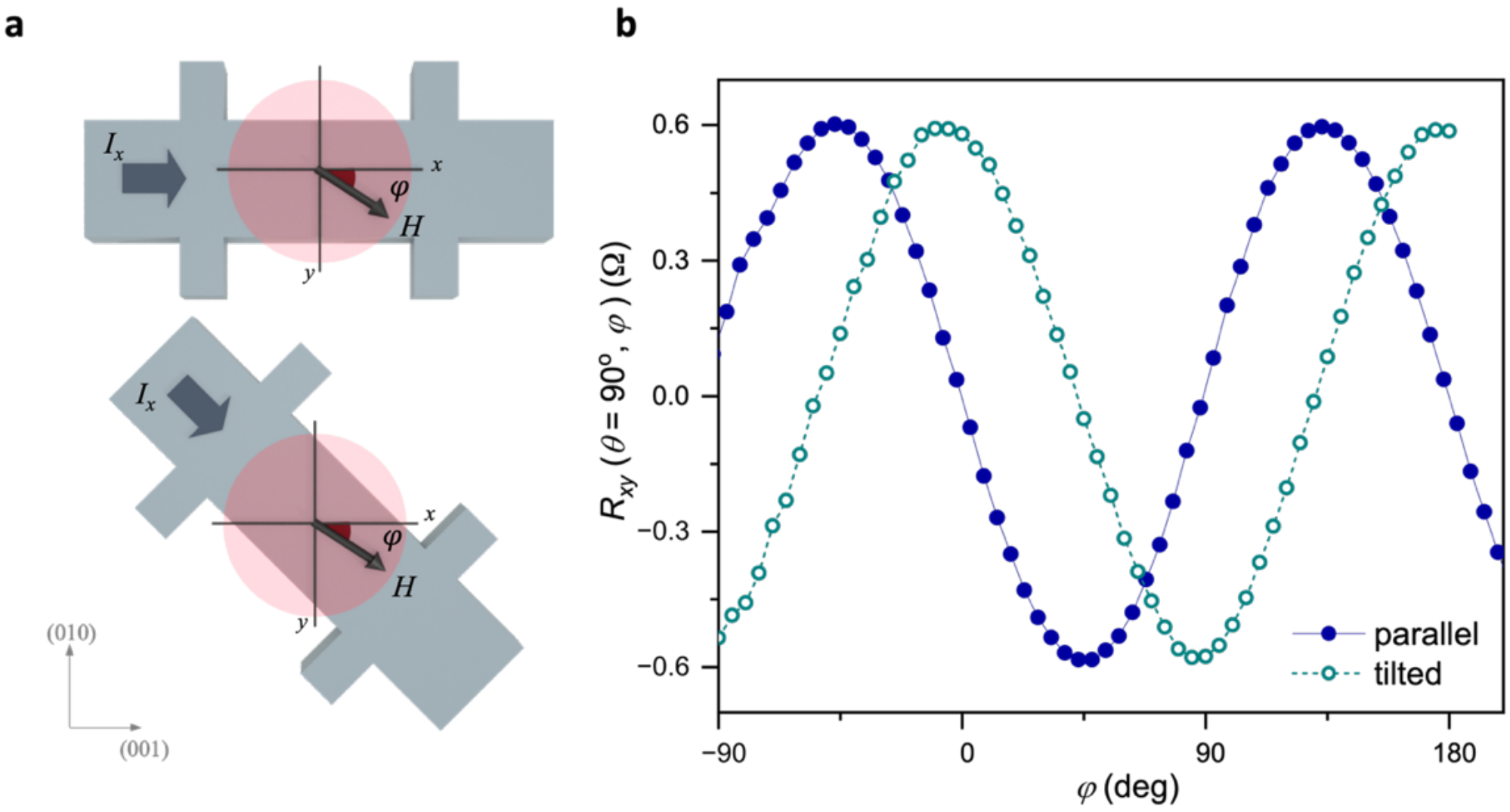

Figure S6- a) Schematic representation of the measurement geometry of two devices patterned with the current direction parallel to the substrate lateral side (top) and at 45º (bottom). b) In-plane angular dependence of the Hall resistance obtained for the device patterned parallel to the substrate side (closed symbols) and at 45º (open symbols), at 88K and 8T.

Figure S6 shows the PHE measurements obtained for two devices patterned along different crystallographic orientations—one with the current parallel to the substrate side and the other at 45º. A clear 45º shift of the signal is observed, which can be attributed to the rotation of the magnetic field axis, independent of the crystal orientation.

S7. Magnetic field dependence of nonlinear transverse resistance at different orientations.

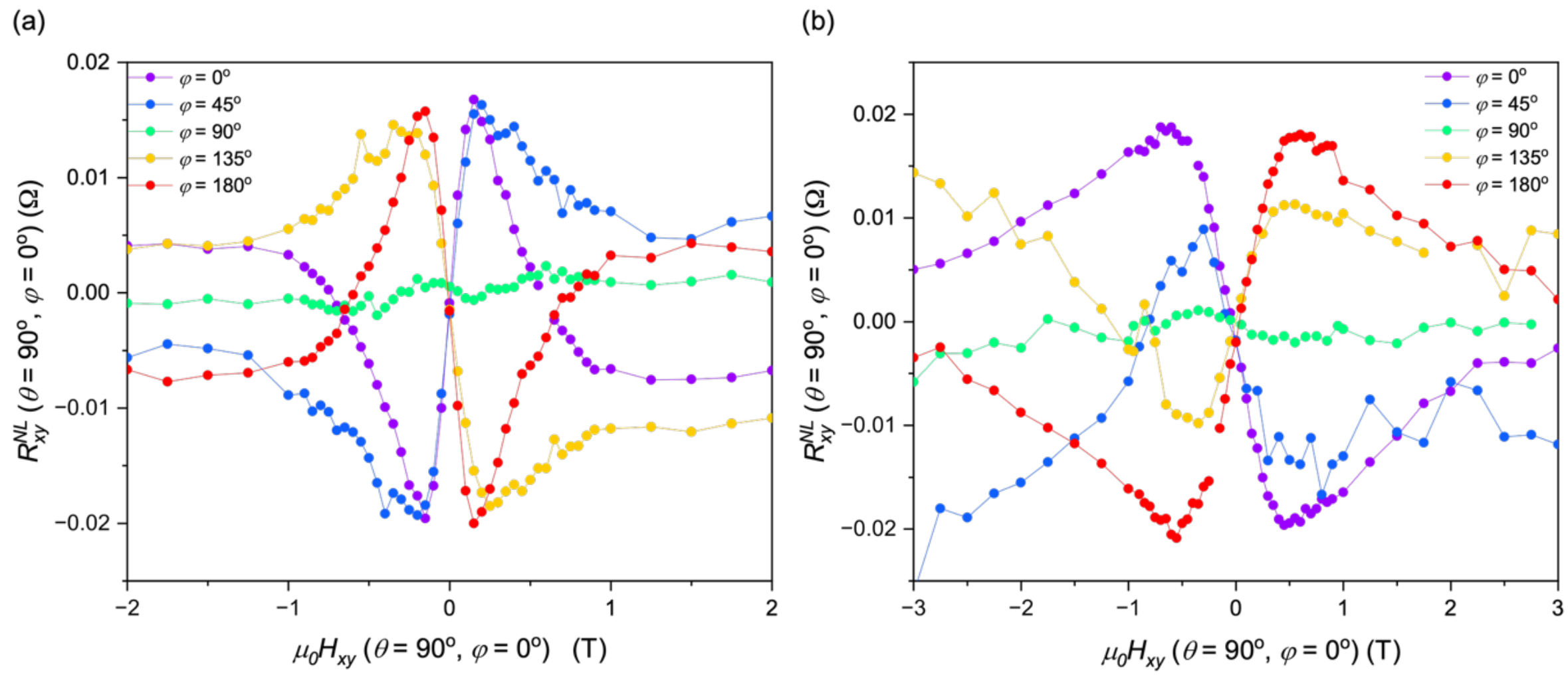

Figure S7- a,b) Magnetic field dependence of the nonlinear transverse resistance measured at 88K (a) and 89K (b), at different in-plane angular orientations.

Figure S7 illustrates the magnetic field dependence of the nonlinear transverse resistance measured at temperatures of 88K and 89K for a 50nm optimally doped device. The resulting curves exhibit a characteristic cos(φ) dependence, revealing distinct maxima and minima at angles of at φ=0º and φ=180º. Notably, a clear sign change is observed between the two temperatures, highlighting the sensitivity of the signal to thermal variations.

S8. Validation of Nonlinear Magnetotransport via Second Harmonic measurements

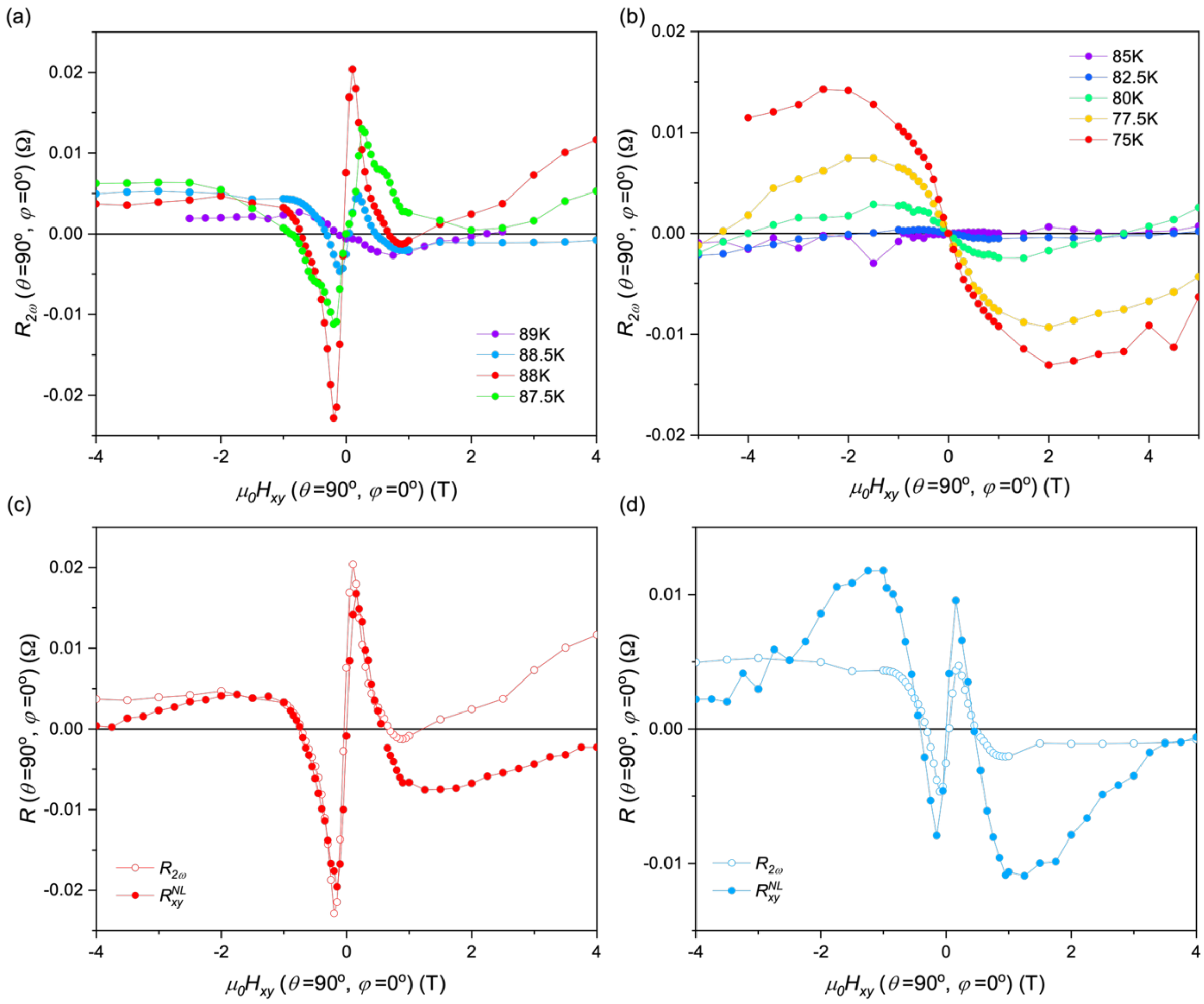

Figure S8- a,b) Second harmonic resistance, $R_{2\omega}$, as a function of in-plane magnetic field applied parallel to the current for the optimally doped and underdoped 50nm YBCO samples, respectively, at different temperatures across the superconducting transition. c, d) Comparison between $R_{2\omega}$ (open symbols) and R_{xy}^{NL} (filled symbols) for two representative temperatures in the optimally doped sample.

Comparison of two approaches for extracting the nonlinear transverse resistance: (i) Second harmonic detection $R_{2\omega}$ using lock-in techniques (Figure S8a,b), and (ii) current-polarity method R_{xy}^{NL} based on the asymmetry between positive and negative currents (Figure 5b,c in the main text). The close agreement between these independent techniques confirms the reproducibility of the nonlinear signal and rules out artefacts associated with a specific measurement protocol, thereby reinforcing the reliability of the main results. The $R_{2\omega}$ measurements were performed using a Zurich Instruments MLFI lock-in amplifier at a frequency of 1000 Hz and an AC current amplitude of 0.3 mA. Temperature and magnetic field control were provided by a Quantum Design PPMS cryostat.
